# Molecular Detection of Human Papillomaviruses in Formalin Fixed Paraffin Embedded Sections from Different Anogenital Lesions in Duhok-Iraq

**DOI:** 10.3390/diagnostics12102496

**Published:** 2022-10-15

**Authors:** Adil Othman, Amer Goreal, Intisar Pity

**Affiliations:** 1 Blood Bank Directorate, Ministry of Health, Kurdistan Region, Duhok P.O. Box 78, Iraq; 2 Department of Medical Microbiology, College of Medicine, University of Duhok, Kurdistan Region, Duhok P.O. Box 78, Iraq; 3 Department of Pathology, College of Medicine, University of Duhok, Kurdistan Region, Duhok P.O. Box 78, Iraq

**Keywords:** human papilloma virus, anogenital lesions, molecular detection

## Abstract

Human Papilloma virus infection is the fundamental reason for the development of ano-genital malignancies and knowing the best tool for diagnostic purposes is mandatory. This study investigated the prevalence and genotype distribution of HPV genotypes in formalin-fixed paraffin-embedded (FFPE) blocks from patients with different anogenital lesions. In this cross-sectional retrospective study, 125 blocks from patients with different anogenital lesions were collected. Three internal sections were taken for HPV detection and genotyping using the paraffin tissue processing kit and HPV Direct Flow CHIP. HPV positivity was detected in 90 (72.0%), with 77 (85.6%) females and 13 (14.4%) males as follows: SCC 64.0%, CINIII 66.7%, CINII 100.0%, CINI 83.3%, KA 83.7%, NILM 44.0%, Anus 66.6%. A total of 44% of histologically unremarkable (negative) cases were positive for HPV genotypes while in only 64% of SCC were HPV genotypes detected. Sixty-six (73.3%) cases were low-risk, and 16 (17.8%) cases were high-risk genotypes, mostly cervical lesions, while seven (7.8%) cases showed a mixed viral detection. The most frequent low-risk genotype was HPV6 genotype (62–68.9%), while the prevalent high-risk HPV was HPV16 genotype (12–13.3%). In this study, HPV16 was more frequently detected than HR-HPV, but mainly in cervical lesion, while HPV6 topped the LR-HPV infections amongst different anogenital lesions in Duhok-Iraq. Higher HPV positivity among cytological unremarkable and abnormal cases may reflect the higher sensitivity of the direct flow CHIP diagnostic technique. The results demonstrate that screening for HPV is essential to reduce cancer development and planning for the vaccine’s introduction in Iraq.

## 1. Introduction

Over 200 genotypes of the Human Papillomavirus (HPV) have been identified according to genomic sequences up to now [1,2]. According to HPV’s association with cancer development, about 60 HPV genotypes that can affect the genital mucosa infect the anogenital area [3]. These viruses are identified as either oncogenic, also called high-risk HPV (HR HPV), or non-oncogenic low-risk HPV (LR HPV) groups based on their association with malignant or benign lesion outcomes such as cervical warts or condylomas [4,5]. The immune system clears most HPV infections on its own, but some infections that persist for several years can have a significant potential to cause tumor malignancy [6,7]. The early oncoproteins (E6 and E7) of HR-HPV strains have oncogenic potential because they can bind to and modulate different gene products, including tumor suppressor proteins (p53 and pRb). As a result of these interactions, cell cycle control is disrupted, and a defect in DNA repair occurs, leading to genomic instability and an increased risk of malignant transformation [8,9]. HPV detection strategies vary in terms of their design and detection targets, which can include HPV DNA or RNA, viral oncoproteins, or other surrogate markers [10,11]. Any HPV detection strategy’s use depends not only on its ability to identify HPV but also on its capability to discriminate between transient and active infections with the potential for malignant transformation [11]. 

Fixation of the specimen with formalin can damage the DNA and can include cross-linking and fragmentation [12]. Amplification of HPV DNA from FFPE specimens is inversely related to the size of the amplicon of the PCR test in addition to the age of specimen, which may enhance degradation [13]. The processing of samples and the extraction of DNA from archival FFPE specimens may have an impact on the sensitivity of different genotyping methods [14]. HPV Direct Flow CHIP is a new commercial tool for highly sensitive HPV detection and genotyping. The direct PCR approach enables for multiplex amplification based on (GP5+/GP6+) from crude cell extracts and hybridization to specific DNA probes to detect high- and low-risk genotypes.

Crude incidence rates of HPV among 100,000 Iraqi people with anogenital lesions have been reported to be low in cancer of the cervix: 1.4, anus: 0.1, vulva: 0.1, and vagina: 0.1 [15]. 

The purpose of the present study was to investigate the prevalence and genotyping distribution of HR-HPV and LR-HPV in ano-genital lesions with different histopathological morphology from archival FFPE blocks.

## 2. Materials and Methods

### 2.1. Study Design and Population

This cross-sectional (retrospective) study was performed on 125 formalin-fixed paraffin-embedded (FFPE) blocks from patients with different anogenital lesions in the period from 2011 to 2021. The samples were retrieved from archives of histopathology departments in Duhok central lab public health and VIN private laboratories. Patients’ ages ranged from 14 to 80 years (Mean: 42 years STD ± 14.6). Females 104 (83.2%) over numbered male cases 21 (16.8%) with 1:4.9 male to female ratio. Lesion locations included 55 (44%) cervix, 36 (28.8%) anus, 21 (16.8%) vulva, 7 (5.6%) vagina and 6 (4.8%) perineum. 

### 2.2. Ethical Approval

Ethical approval to conduct the study was obtained from the Research Ethics Committee at Duhok Directorate General of Health, at 20 July 2020-Reference number (20072020-3) 

### 2.3. Sample Collection 

The biopsies which were fixed in FFPE were sectioned. Five serial sections with 10 µm thick using a new blade between each sample to avoid cross-contamination were taken from each biopsy, two outer sections were stained with Hematoxylin and Eosin, examined under a microscope to confirm the diagnosis that classified as: Unremarkable (non-neoplastic) pathology; Negative for Intraepithelial Lesions or Malignancy (NILM); Koilocytotic atypia (KA); Cervical Intraepithelial Neoplasia (CIN) grade 1 (CIN1); CIN grade 2 (CIN2); CIN grade 3 (CIN3); and Squamous Cell Carcinoma (SCC).

### 2.4. DNA Extraction

Using a paraffin tissue processing kit (Master Diagnostica, Granada, Spain), for HPV DNA extraction, three internal sections were placed in 1.5 mL an Eppendorf tube and volume 400 µL of mineral oil were added to the sample, heated at 95 °C for 2 min then centrifuge for 2 min at 2000 rpm and the remains of mineral oil were removed. Sixty µL of Extraction Buffer and 1.5 µL of DNA Release were added. The mixture was incubated for 30 min at 60 °C, followed by 10 min at 98 ° C in a thermal block, and then centrifuged for 2 min at 2000 rpm, and 4–6 µL of the homogeneous suspension as a DNA template taken for the PCR reaction.

### 2.5. HPV Detection and Genotyping

Detection and genotyping HPV by the HPV Direct Flow Chip (Master Diagnostica, Granada, Spain) is designed to simultaneously screen and genotype 36 HPV types, 18 high-risk HPV (16, 18, 26, 31, 33, 35, 39, 45, 51, 52, 53, 56, 58, 59, 66, 68, 73 and 82) and 18 low-risk HPV (6, 11, 40, 42, 43, 44, 54, 55, 61, 62, 67, 69, 70, 71, 72, 81, 84, and 89). This technique is based on PCR amplification of the L1 consensus region of the human papilloma virus, that includes amplification of a 268 bp fragment (internal control) of the human beta globin gene and a 150 bp fragment (GP5+/GP6+) of the HPV L1 region, then hybridization to specific immobilized DNA probes on a nylon membrane. A DNA flow-based automatic and manual hybriSpot platforms allows the amplified DNA to bind to complementary specific probes in a three-dimensional porous environment. Biotinylated PCR products are hybridized with specific probes and the hybridization signals are developed by colorimetric immunoenzymatic reaction with streptavidin–alkaline phosphatase and a chromogen (NBT/BCIP), generating insoluble precipitate in the position of the membrane where the specific probe has hybridized with the PCR amplicon, and this signal is automatically captured and analyzed with the hybriSoft™ software-VIT-HS12-VITRO, S.A. Sevilla, Spain.

### 2.6. Statistical Analysis

Data were collected, revised, and entered using IBM SPSS statistics version 25. Chi-Square (χ2) test was used to find the associations among categorical parameters, and differences at the level of *p* ≤ 0.05 were considered as statistically significant.

## 3. Results

One hundred and twenty-five cases of anogenital lesions were described. Patients’ ages ranged from 14 to 80 years (mean: 42 years STD ± 14.6). Females 104 (83.2%) over numbered male cases 21 (16.8%) with 1:4.9 male to female ratio. Lesion locations included 55 (44%) cervix, 36 (28.8%) anus, 21 (16.8%) vulva, 7 (5.6%) vagina and 6 (4.8%) perineum. Morphologically, 49 (39.2%) showed Koilocytotic atypia (KA), 25 (20.0%) unremarkable (non-neoplastic) pathology negative for intraepithelial lesions or malignancy (NILM), 25 (20.0%) squamous cell carcinoma (SCC) malignant, 18 (14.4%) cervical intraepithelial neoplasia (CIN1), 5 (4.0%) CIN2, and 3 (2.4%) CIN3. Using HPV Direct Flow CHIP, HPV positivity was detected in 90 (72.0%) patients, with 77 (85.6%) females and 13 (14.4%) males. There were no age- or gender- significant differences (*p* = 0.192). HPV DNA was identified in 44.0% of unremarkable (negative) cases and 79.0% of abnormal morphology cases. HPV DNA dominant positivity was identified in KA 41 (45.6%), followed by SCC 16 (17.8%), CIN1 15 (16.7%), NILM 11 (12.2%), CIN2 5 (5.6%), then CIN3 (2.2%). The differences were highly significant. HPV genotype positivity was demonstrated in 100% of CIN2, 83.7% of KA, 83.3% of CIN1, 66.7% of CIN3 and 64% of SCC (Table 1).

As shown in (Table 2), although statistically not significant, the third and fourth decades dominated the low-risk group (40.0%), whereas the fourth and fifth decades dominated the HR-HPV types (13.4%). Of the positive cases, 66 (73.3%) cases were low-risk, and 16 (17.8%) cases were high-risk genotypes, seven (7.8%) cases showed mixed viral detection. In a single positive case, no definite genotype could be identified (Table 2). 100% of positive CIN3 cases were of HR-HPV, equal frequency, low- and high-risk genotypes in CIN2, but more dominant LR-HPV DNA among the remainders. 

**Figure 1:**
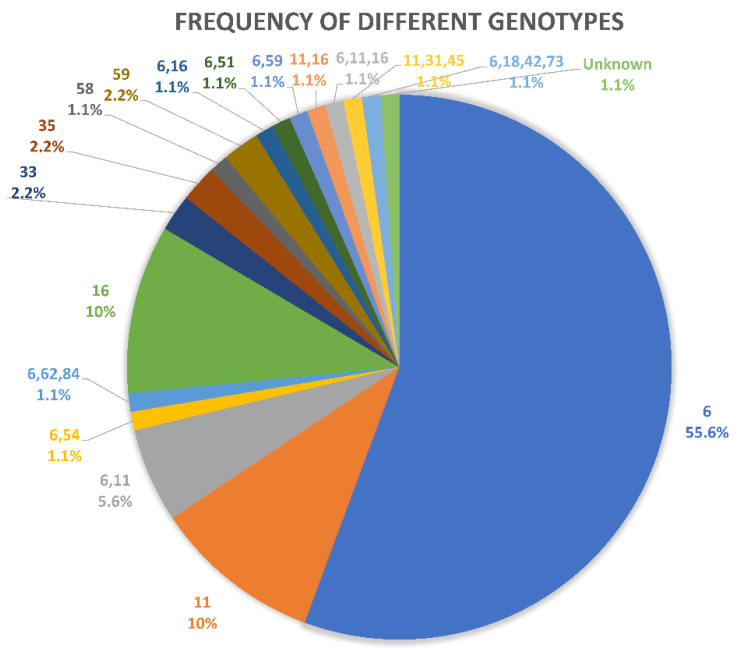
Frequency of HR-HPV & LR-HPV and Mixed groups.

As demonstrated in (Figure 1), the most frequent, 62 (68.9%), low-risk genotypes were HPV6, while the prevalent, 12 (13.3%), HR-HPV was HPV 16 genotype (single and mixed). χ^2^ test used, *p* = 0.000.

**Figure 2:**
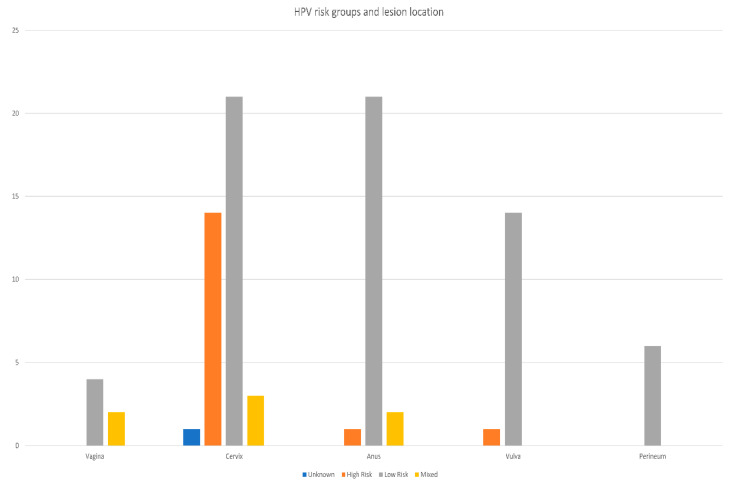
HPV risk groups in relation to anatomical location.

Considering the location, the most frequent LR-HPV genotypes were described in the cervix and anus (31.8% each), and the cervix dominated the HR-HPV (87.5%) and mixed genotype (42.9%), (Figure 2), χ^2^ test used, *p* = 0.015.

**Figure 3:**
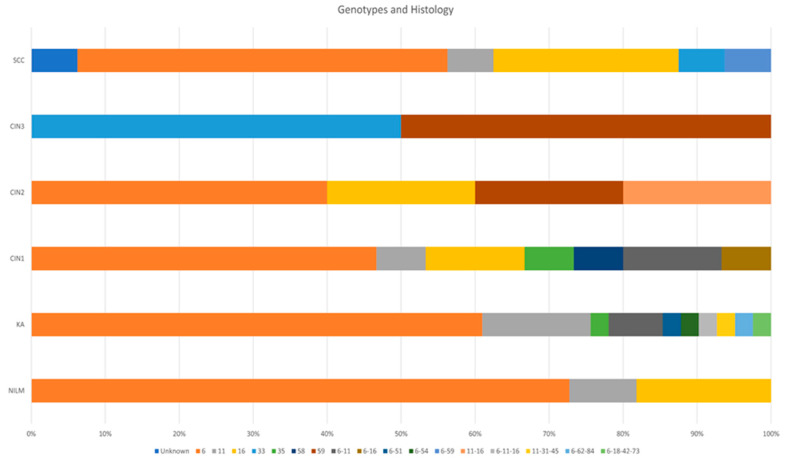
HPV genotypes in relation to histopathological diagnosis.

As shown in (Figure 3), the most frequent HPV genotypes (single and mixed) in Koilocytotic atypia were HPV6 (n = 33; 80.4%) and HPV11 (n = 11; 26.8%), in cancer cases HPV6 (n = 9; 56.2%) and HPV16 (n = 4; 25.0%), in CIN1 HPV6 (n = 10; 66.6%), HPV11 (n = 3; 20.0%) and HPV16 (n = 3; 20.0%), in NILM HPV6 (n = 8; 72.7%) and HPV16 (n = 2; 18.1%), CIN2 HPV6 (n = 2; 40.0%) and HPV16 (n = 2; 40.0%), Intraepithelial neoplasia were HPV6 (n = 11; 45.8%) and HPV16 (n = 3; 12.5%), then in CIN3 HPV33 (n = 1; 50.0%) and HPV59 (n = 2; 50.0%). 

Among HPV DNA positive cases, 75 (83.30%) cases were a single genotype infection, 10 (11.10%) dual infection, 3 (3.30%) triple infections, 1 (1.10%) with quadrant infection and 1 (1.10%) with undetectable genotype, χ^2^ test used, *p* = 0.015.

## 4. Discussion

Few studies related to the molecular detection of Human Papillomavirus (HPV) have been established in Iraq including Duhok Governorate, most have used the conventional PCR and strip test with special concentration on HPV16 and (or) 18 high-risk genotypes. In the present study, PCR molecular tool and HPV Direct Flow CHIP assay for identification of HPV genotypes (low and high-risk) were applied for detection of different HPV genotype DNA on formalin-fixed, paraffin-embedded (FFPE) histologically diagnosed tissue samples. Among 125 samples, 72.0% were positive for HPV genotype. Such a frequency rate is compatible with other studies performed in Baghdad/Iraq, Jordan and Cyprus [16,17,18]. However, higher and lower rates have been observed in different geographic states.

The lower rates of HPV described in some countries, despite the close religious and socioeconomic status, can be related to the use of different commercial DNA purification kits, as some may cause degradation and cross-linking, leading to low intra-assay reproducibility [19,20,21]. Considering the age and sex, although statistically not significant, higher HPV rates were observed among young women (21–40 years of age). Such observation is considered logical because of the prevalence of HPV infection during the reproductive-aged women. However, most HPV infections are found to be cleared over a period of 6–12 months, and only a small proportion develops a persistent infection [22]. Several researchers observed HPV infection rates to be higher in age groups even younger than 20 years of age [23]. Such higher viral prevalence among young women may be related to their early sexual activity [24,25]. Religion and cultural upbringing of the Iraqi people may play a role in protecting them from earlier infection. Considering different morphologies, HPV DNA was identified in 44.0% of unremarkable (negative) cases and 79.0% of abnormal morphology cases. In the study done in Duhok –Iraq in 2019, 46.2% of negative Pap smears and 53.8% of abnormal Pap cases were positive for HPV [26]. In contrast, a study in Erbil/Iraq reported that no HPV was found in negative cervical specimens and 71% in abnormal biopsy results [27]. Our higher HPV positivity among cytologically unremarkable and abnormal cases in the current study compared with the mentioned two local studies may reflect the higher sensitivity of the Direct Flow CHIP methodology applied in this study in spite of the fact that a high percentage of LR-HPV in anal samples may have contributed to the results. Heterogeneous results obtained in different neighboring countries such as Saudi Arabia and Kuwait were (28.6%), (36.8) in normal and (53.1%) and (63.2%) of abnormal morphology, respectively. [28,29]. The high-risk (HR-HPV) positivity in this study (17.8%) was close to what has been reported previously in a study in the same locality [30] and in a study in Iran [31]. However, higher and lower HR-HPV frequency rates have been described in different geographic studies [17,18,32]. Sample size, sample use and the techniques applied play great roles in such heterogeneous results. Amongst positive HPV genotypes, HPV16, whether pure or mixed with others, was the dominant high-risk genotype (13.3%). Although more or less similar findings have been reported in several local and international studies [26,31,33], lower rates have been described by others [19,34]. In Baghdad/ Iraq, HPV33 topped their list [34], while in Saudi Arabia, HPV68 was dominant, followed by HPV 18 and 16 [19]. Low-Risk HPV (LR-HPV) DNA positivity in this study formed 73.3%, in which HPV6 (68.9%) topped the list followed by (single and mixed) HPV11 (14.5%). In parallel, Iran studies described HPV6 and HPV11 as the top two prevalent LR-HPV genotypes [31]. However, the most frequent LR-HPV genotype in Qatar was HPV81 [20], Such variations in high- and low-risk distribution can be explained by geographic diversity and sample sizes in addition to the selected tested people. In the present study, HPV6 formed the commonest finding in both unremarkable (72.7%) and morphologically abnormal cases, including koilocytosis (61.0%), SCC (50.0%) and CIN1 (46.7%). Most positive cases were identified amongst CIN2 (100%), koilocytotic atypia and CIN1 (83.7% and 83.3%), respectively, followed by CIN3 (66.7%) and SCC (64%). Although the rate of anal cancer is increasing due to changes in sexual behaviors, with persistent anal HPV infection being the major cause of anal cancer [35], HR-HPV was mainly detected in cervical lesions rather than anal ones.

Negative viral genotypes in neoplastic cases may be caused by a real absence of the virus due to the local traditions in addition to the increase in vaccinations in recent decades. False negative results owing to the fixative used, and the processing system applied, may also contribute to negative HPV DNA identification. As well, the decreasing frequency of HR-HPV genotypes in cancer cases can be explained by the fact that the viral impact is an early event in oncogenesis, and with increasing progression, other oncogenes may contribute to the oncogenesis process, so that viral infection is enrolled in the development of cancer initially but later resolved. In addition, the integration of HPV DNA in cervical carcinoma may have disrupted PCR primer target sequences or resulted in loss of the L1 open reading frame. Furthermore, HR-HPV vaccination and women’s education may decrease the positive predictive HR-HPV values, a concept that is strengthened by decreasing HR groups amongst the low grades of CIN in addition to KA and negative cases. Among our positive cases, a single HPV genotype was identified in 83.3%, while in the remainders (15.6%), mixed viral genotype infections were described. Such rates appear parallel with what have been obtained in previous studies performed on 64 Duhok/Iraq females [26] and 856 Bagdad/Iraq females [34], and are lower than what has been previously reported in another Baghdad study performed on 100 FFPE tissue samples [16], and 209 Pap smear samples in Jordan [17]. The types of people examined, with their different social habits and geographic distribution, in addition to the samples (fluid or tissue) examined, and the technique applied for HPV detection, may impact the results. There is no consensus on the association of multiple infections with occurrence or progression of cervical cancer. For instance, Fife et al. showed that multiple HR-HPV infections tended to increase the risk of cervical diseases [36], while Jung et al. found that multiple infections were less frequently associated with cervical neoplasia [37]. According to the one virus one lesion hypothesis, it seems unlikely that several different HPV genotypes infect the same cell, but that each one is associated with a different lesion [38]. Such findings might provide a reference for future HPV-based anogenital cancer screening tests, treatment of HPV infection and the application of other HPV genotype vaccinations in Iraq.

The study’s strength is that this is the first time in the region that HPV-DNA was extracted from archival FFPE specimens many years old, with full histopathological reports.

Limitations of the study included fragmentation of DNA in some old specimens, and the low number of anal specimens available.

## 5. Conclusions

In this study, HPV16 and HPV6 topped the HR-HPV and LR-HPV infections, respectively, amongst different anogenital lesions in Duhok-Iraq. Decreasing HR-HPV frequencies in cancer and low-grade CIN cases may be contributing to viral impact as an early event in oncogenesis, the integration of HPV DNA in cervical cancer may give disrupted PCR primer target sequences or result in the loss of the L1 region. Higher HPV positivity among cytologically unremarkable and abnormal cases in the current study may reflect the higher sensitivity of the Direct Flow CHIP kit methodology that is suitable for screening, although the high positivity of LR-HPV in anal lesions may have had an impact on the results. It is fundamental to start planning to implement HPV vaccination in Iraq.

## Figures and Tables

**Table 1:** Positive-negative HPV with histopathological morphology.

Result		NILM	KA	CIN1	CIN2	CIN3	SCC	Total
Positive	Count	11	41	15	5	2	16	90
	% within Result	12.2%	45.6%	16.7%	5.6%	2.2%	17.8%	100.0%
	% within Histology Diagnosis	44.0%	83.7%	83.3%	100.0%	66.7%	64.0%	72.0%
	% of Total	8.8%	32.8%	12.0%	4.0%	1.6%	12.8%	72.0%
Negative	Count	14	8	3	0	1	9	35
	% within Result	40.0%	22.9%	8.6%	0.0%	2.9%	25.7%	100.0%
	% within Histology Diagnosis	56.0%	16.3%	16.7%	0.0%	33.3%	36.0%	28.0%
	% of Total	11.2%	6.4%	2.4%	0.0%	0.8%	7.2%	28.0%
Total	Count	25	49	18	5	3	25	125
	% within Result	20.0%	39.2%	14.4%	4.0%	2.4%	20.0%	100.0%
	% within Histology Diagnosis	100.0%	100.0%	100.0%	100.0%	100.0%	100.0%	100.0%
	% of Total	20.0%	39.2%	14.4%	4.0%	2.4%	20.0%	100.0%

χ^2^ test used, *p* = 0.005.

**Table 2:** High and Low-risk HPV risk in relation to different age groups.

Age Groups	Risk Group				
	Unknown	High-Risk	Low-Risk	Mixed	Total
11–20	0 (0.0%)	0 (0.0%)	3 (3.3%)	1 (1.1%)	4 (4.4%)
21–30	0 (0.0%)	2 (2.2%)	18 (20.0%)	2 (2.2%)	22 (24.4%)
31–40	0 (0.0%)	6 (6.7%)	18 (20.0%)	1 (1.1%)	25 (27.8%)
41–50	0 (0.0%)	6 (6.7%)	13 (14.4%)	2 (2.2%)	21 (23.3%)
51–60	1 (1.1%)	0 (0.0%)	9 (10.0%)	0 (0.0%)	10 (11.1%)
61–70	0 (0.0%)	2 (2.2%)	3 (3.3%)	1 (1.1%)	6 (6.7%)
71–80	0 (0.0%)	0 (0.0%)	2 (2.2%)	0 (0.0%)	2 (2.2%)
Total	1 (1.1%)	16 (17.8%)	66 (73.3%)	7 (7.8%)	90 (100.0%)

χ^2^ test used, *p* = 0.345.

## Data Availability

All data are available within the text.
